# P-286. Regional Variation in Hospitalization Trends Among Insured Persons with HIV in the United States, 2017–2020

**DOI:** 10.1093/ofid/ofaf695.507

**Published:** 2026-01-11

**Authors:** Tonisha Gaitor, Daniel B Chastain, Xianyan Chen, Savannah M Hammerton

**Affiliations:** University of Georgia College of Pharmacy, Augusta, GA; University of Georgia College of Pharmacy, Augusta, GA; UGA Franklin College of Arts and Sciences, Athens, Georgia; University of Georgia, Athens, Georgia

## Abstract

**Background:**

Hospitalization rates among persons with HIV (PWH) in the US remain substantial, yet regional variations are understudied. This study analyzed insurance claims data to estimate hospitalization incidence among commercially and Medicare-insured PWH across four US regions.

**Figure 1.**
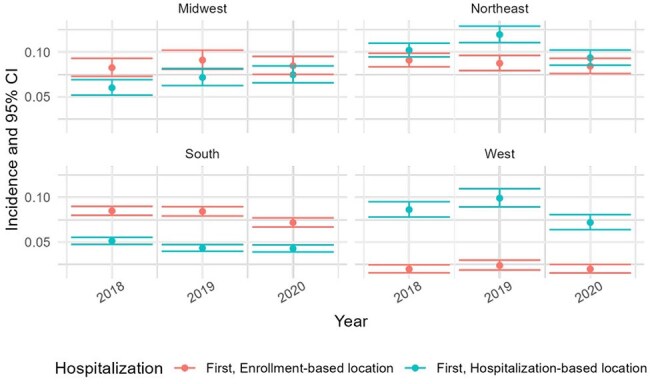
Incidence proportion of persons with more than one hospitalization

The proportion of PWH with ≥1 hospitalization annually was calculated by dividing the number of individuals with ≥1 hospitalization by the total number of individuals with HIV at the beginning of each year. Rates are shown stratified by region of enrollment (red points and error bars) and admitting hospital region (blue points and error bars). From 2018 to 2020, enrollment-based rates were highest in the Northeast and lowest in the West. The Northeast consistently had the highest incidence (0.09 [95% CI: 0.08–0.10] in 2018 and 2019), while the West remained lowest (0.02 [95% CI: 0.02–0.02] in 2018 and 0.02 [95% CI: 0.02–0.03] in 2019 and 2020). In 2020, a slight decrease was observed in the South (from 0.08 to 0.07), while the Northeast and Midwest remained stable. Hospital-based stratification showed a slightly different pattern: the Northeast remained highest, peaking at 0.12 (95% CI: 0.11–0.13) in 2019, while the South had the lowest proportions, decreasing from 0.05 (95% CI: 0.05–0.06) in 2018 to 0.04 (95% CI: 0.04–0.05) in 2020. The West and Midwest showed modest fluctuations, with the West peaking at 0.10 (95% CI: 0.09–0.11) in 2019 before declining to 0.07 (95% CI: 0.06–0.08) in 2020.

**Figure 2.**
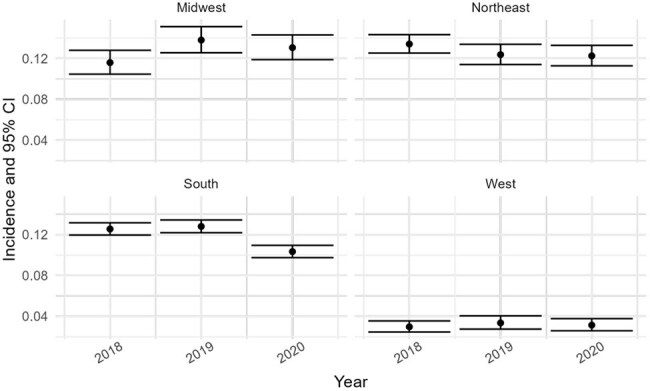
Incidence proportion of all hospitalizations B. Incidence proportion of all hospitalizations.

Total hospitalizations per regional person-years was calculated by dividing the number of hospitalizations by the total number of individuals with HIV at the beginning of each year. Results are stratified by enrollment region (black points and error bars). From 2018 to 2020, the highest incidence proportions were observed in the Northeast and South (range: 0.12–0.13). The Midwest increased from 0.12 in 2018 to 0.14 in 2019, then declined slightly in 2020. The West consistently had the lowest incidence, remaining at 0.03 each year with narrow confidence intervals.

**Methods:**

This study used MarketScan data (2017-2020) to determine hospitalization incidence in US PWH (ICD-10-CM B20), using 2017 to identify cases for 2018. To ensure complete annual hospitalization capture and minimize loss-to-follow-up bias, the cohort was limited to continuously enrolled individuals (full 12-month coverage/year). For regional analysis across four US Census regions (South, Northeast, Midwest, West, encompassing all 50 states and D.C.), the population was further restricted to enrollees with available regional data. Annual hospitalization incidence was reported as (1) incidence proportion of ≥1 hospitalization, (2) incidence proportion of all hospitalizations, and (3) incidence density, each with 95% confidence intervals.

**Figure 3.**
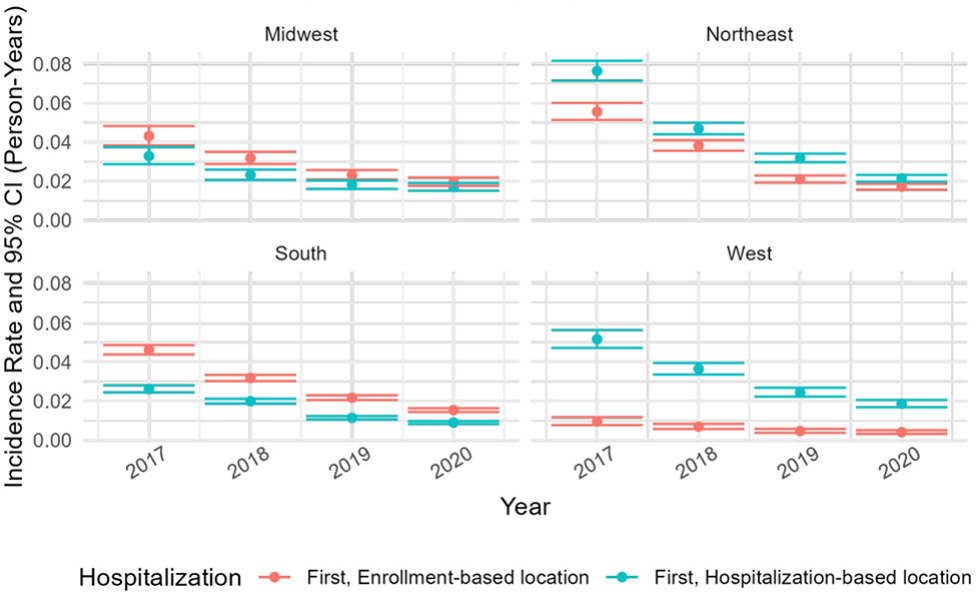
Incidence density of first hospitalization

Incidence density was defined as the number of individuals with a first hospitalization divided by the total person-time at risk (measured as the sum of days prior to first hospitalization or, if no admission occurred, the total follow-up time). Results are stratified by enrollment region (red points and error bars) and hospital region (blue points and error bars). Incidence density declined across all regions from 2018 to 2020. In enrollment-based analyses, the Northeast had the highest rates, dropping from 0.038 (95% CI: 0.036–0.041) in 2018 to 0.017 (95% CI: 0.016–0.019) in 2020. The West had the lowest rates, declining from 0.007 (95% CI: 0.006–0.008) to 0.004 (95% CI: 0.003–0.005). Hospital-based rates followed similar regional trends, with the Northeast consistently highest. While the South and West showed lower rates overall, West region estimates were slightly higher than those based on enrollment.

**Results:**

Among 90,836 PWH, 13.5% (n=12,287) had ≥1 hospitalization, a stable proportion. Hospitalized individuals were typically 49–50 years old (median), 73% male, and 95% commercially insured, with most experiencing ≥2 annual hospitalizations (3–4-day median stay). The South (39%) and Northeast (29%) accounted for the largest, stable proportions of all hospitalizations. Hospital-based incidence mirrored enrollment trends but showed greater variation, peaking in the Northeast and declining in the South (Fig). Total hospitalization incidence proportions were higher than ≥1 hospitalization proportions, reflecting multiple admissions. Incidence density revealed temporal declines and regional nuances, suggesting reduced hospitalization frequency in the Northeast and South despite stable proportions, and consistently low rates in the West.

**Conclusion:**

Hospitalization patterns varied regionally, with the highest burden in the Northeast and South. While the proportion of individuals hospitalized remained stable, declining incidence density in these regions suggests reduced hospitalization frequency over time. These findings emphasize the need to explore factors such as access to care and comorbidities driving regional disparities.

**Disclosures:**

All Authors: No reported disclosures

